# Psychosocial risk and job satisfaction in professional drivers

**DOI:** 10.3389/fpsyg.2022.994358

**Published:** 2022-09-07

**Authors:** Víctor Hernández-Rodríguez, Elvira Maeso-González, Mario Gutiérrez-Bedmar, Antonio García-Rodríguez

**Affiliations:** ^1^Research Group Work and Transportation Management, School of Industrial Engineering, University of Malaga, Malaga, Spain; ^2^Department of Economics and Business Management, University of Malaga, Malaga, Spain; ^3^Biomedical Research Institute of Malaga-IBIMA, Malaga, Spain; ^4^Department of Public Health and Psychiatry, School of Medicine, University of Malaga, Malaga, Spain; ^5^CIBERCV Cardiovascular Diseases, Carlos III Health Institute, Madrid, Spain

**Keywords:** psychosocial risk, stress, job satisfaction, health occupational, professional drivers, COPSOQ

## Abstract

Psychosocial work factors are implicated in the development of stress and job satisfaction. This relationship has been scarcely studied in so-called singular activities, as is the case of professional drivers. This cross-sectional study of 601 professional drivers assesses psychosocial risks and job satisfaction using Copenhagen Psychosocial Questionnaire (COPSOQ) and Overall Job Satisfaction questionnaire models. The values of the psychosocial scales of professional drivers were compared with thresholds values of the Spanish working population. The relationships of the psychosocial scales with the level of job satisfaction were examined using logistic regression models, adjusted for age, gender, length of driving license and years of experience. In general, professional drivers have an unfavorable psychosocial environment compared to the average Spanish workers. The relationship between psychosocial scales and job satisfaction is observed, with the most influential variables being the meaning and development of work, integration in the company, social relations, esteem and level of psychological demands.

## Introduction

Industrialized countries have to face major challenges related to psychosocial aspects of work as a result of the significant increase in workload and work stress (Nübling et al., 2006). Psychosocial risks and work-related stress are among the most difficult problems in occupational safety and health (Hassard and Cox, 2015; Kresal et al., 2017; Mościcka-Teske et al., 2019). The Enterprise Survey on New and Emerging Risks (ESENER, 2019) shows that psychosocial risks are considered more challenging and more difficult to manage than “traditional” occupational safety and health risks. This fact, which is increasingly recognized in large companies, is, however, more difficult to address in particular, but very frequent, professions, such as professional drivers.

Psychosocial risk at work is defined as “those aspects of the design, organization and management of work, as well as its social and environmental context, which have the potential to cause physical, psychological or social harm to workers” (Cox et al., 2005). On the other hand, when the balance between individual factors and working conditions is not achieved in an organization, situations of job dissatisfaction like irritation, tension, depression or reduced ability to concentrate, arise (Royo et al., 2016). It is therefore interesting to relate job satisfaction with psychosocial risk (Briones et al., 2010).

Previous studies have analyzed in depth the psychosocial burden in classic sectors such as teaching (Galdeano et al., 2007; Gutiérrez-Bedmar et al., 2009), healthcare (Stoddard et al., 2001; Hamdan-Mansour et al., 2011; García-Rodríguez et al., 2015; Miller et al., 2018), and social workers (Rodriguez-Alonso et al., 2017; Rivera-Rojas et al., 2021), profiles that are characterized by suffering a higher degree of stress. However, studies in unique sectors are scarce. Some studies can be found in miners (Mościcka-Teske et al., 2019), in female seasonal workers (Palomo-Vélez et al., 2015) and in Professional Drivers but in this case related to specific diseases (Kresal et al., 2017; Wu et al., 2019).

In Spain about half a million people are professional drivers (DGT, 2018). In addition, many people make driving their usual working livelihood, so, the impact of this activity for the health and behavior of workers can be very important (Fundación CEA, 2016).

The first aim of the present study was to analyze psychosocial factors in the usual work of professional drivers and to compare them with the general working population. A second objective was to relate psychosocial risk and job satisfaction of professional drivers.

## Materials and methods

For the assessment of psychosocial factors at work we used the Copenhagen Psychosocial Questionnaire (COPSOQ). This tool was originally developed at the National Institute of Occupational Health in Denmark by Kristensen and Borg in 2000 (Kristensen et al., 2005), and was later adapted and validated in Spain as Istas21 (Moncada-Lluís et al., 2008). The reference levels of the psychosocial scales of the Spanish salaried population have been obtained from the last one. Copenhagen Psychosocial Questionnaire is designed to assess any type of economic activity, can be used in any workplace and its ultimate aim is to obtain a healthier work organization. In a recent study, a complete description of the validation of the measurement of psychosocial factors at work in professional drivers has been carried out using the COPSOQ-III version in professional drivers, using the COPSOQ-III version (Useche et al., 2019).

**Figure 1:**
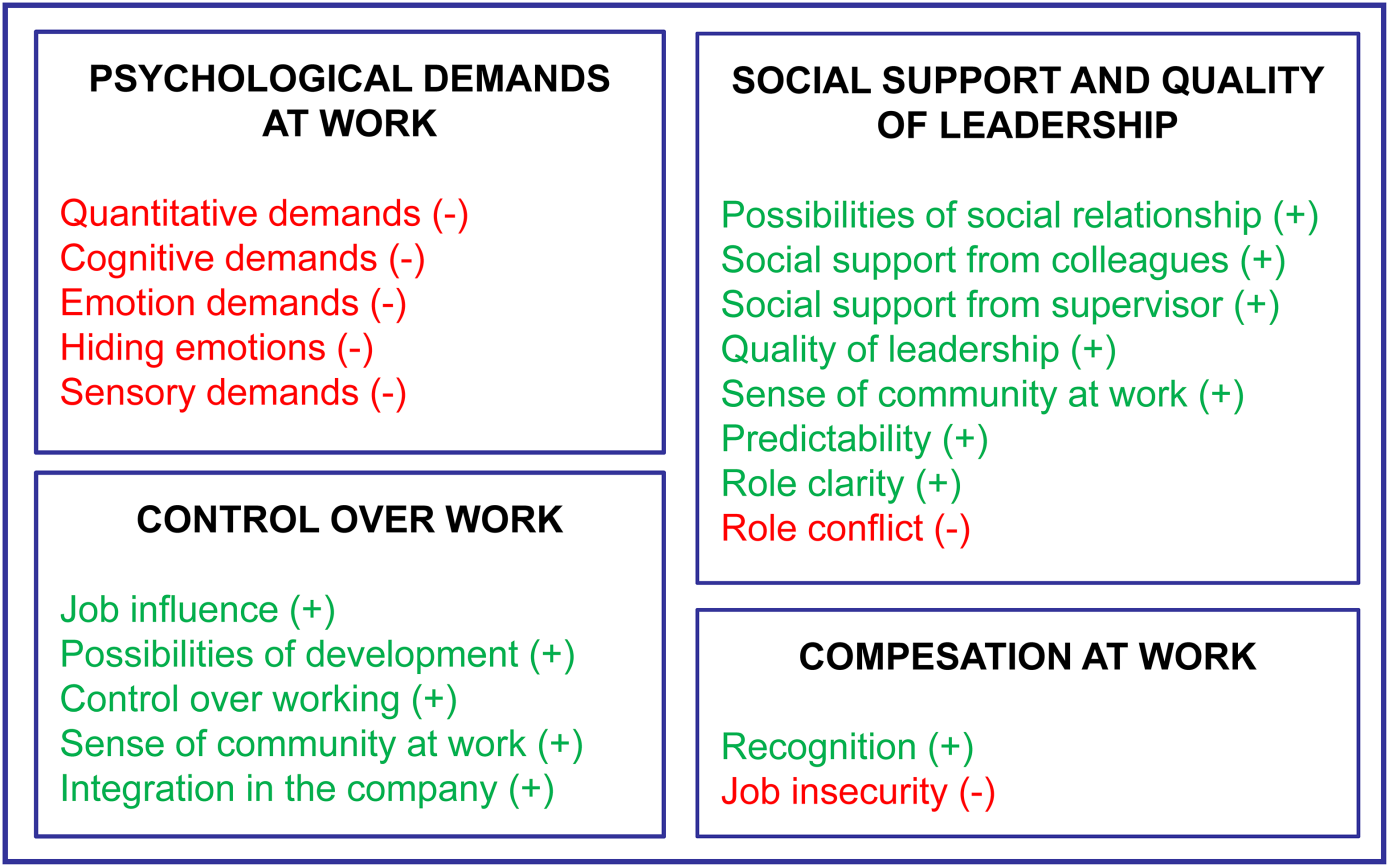
Psychosocial dimensions of the COPSOQ questionnaire.

Copenhagen Psychosocial Questionnaire covers the main areas of the psychosocial work environment: demands at work, work organization and interpersonal relations. The instrument includes 20 scales grouped in the following sections: Psychological demands at work; Control over work, influence, and development; Social support and quality of leadership; and Degree of compensation at work. The Figure 1 shows the content of the COPSOQ questionnaire. We have differentiated the scales into two main groups: positive scales (marked in green) and negative scales (red). The positive scales are those that reflect positive aspects of the work organization and therefore, a high score on these scales implies a good situation for the worker. On the other hand, negative scales summarize work conditions that are detrimental to the worker’s health and therefore a high score on these scales implies a risky situation for the worker. Due to the characteristics of the work environment and the type of activity performed by our participants, we only used 16 COPSOQ scales of the medium-length version to measure the psychosocial work environment. These scales consist of 56 different Likert-type items. Scales values were calculated by summing up the numerical values attached to the response categories of the items. All the scales were transformed to a range from 0 to 100. Directions of the scores follow the label of the scale; i.e. a high score on quantitative demands scale indicates high quantitative demands, a high score on the possibilities for development indicates high possibilities for development, and so on. A detailed description of the items and the scales is available elsewhere in the literature (Kristensen et al., 2005), and on the internet.^1^

**Figure 2:**
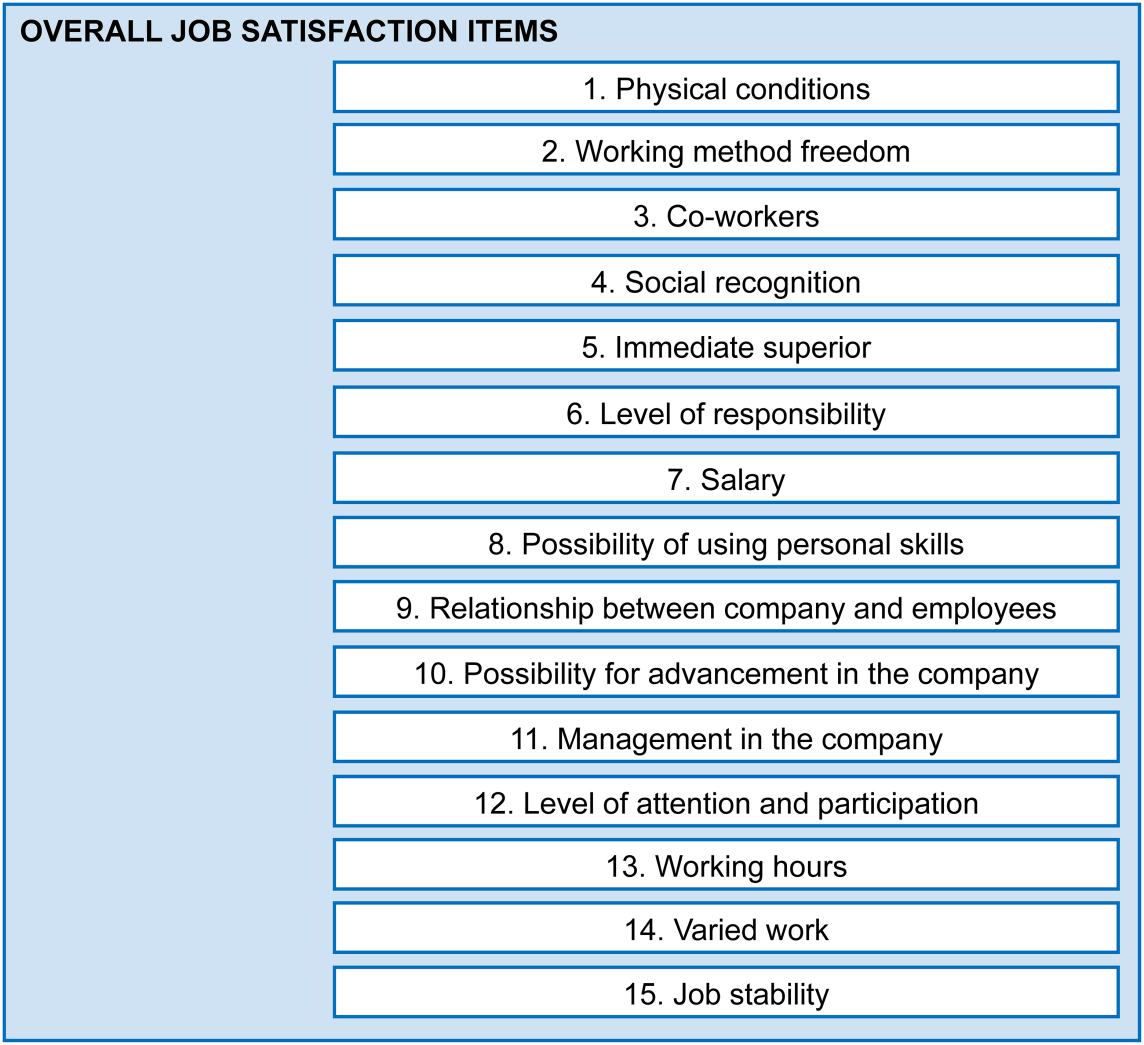
Description of the job satisfaction items (Overall).

To measure job satisfaction, we used the “Overall Job Satisfaction Questionnaire” (Warr et al., 1979), which comprises 15 items described in Figure 2. It is a tool for identifying job satisfaction of workers in paid employment, in three dimensions: general, extrinsic, and intrinsic. It is an additive Likert-type scale where the total score is obtained from the sum of the respondent’s rankings on each of the 15 items, assigning a value of 1 to “very dissatisfied” and, correlatively, until a value of 7 is assigned to “very satisfied.” All items are single response ordinal qualitative variables, consequently, the total score ranges between 15 and 105, where the higher the score the higher the job satisfaction. Items 1, 3, 5, 7, 9, 11, 13, and 15 assess aspects related to extrinsic satisfaction and items 2, 4, 6, 8, 10, 12, and 14 measure intrinsic employee satisfaction.

This is a cross-sectional study in which the study population is professional drivers in Spain. The sample size, 385 surveys, was estimated for an infinite population, with a degree of heterogeneity or diversity of 50% (*p* = *q*), a confidence level of 95% and a margin of error or precision of 5% (*e* = 5). Drivers taking training and refresher courses at 12 training centers in different Spanish provinces were invited to take part in the study. Informative talks were given beforehand with the drivers and the managers of the centers. Drivers agreed to take part, sending the questionnaires by post in sealed envelopes, guaranteeing confidentiality and anonymity at all times.

The statistical analysis included a descriptive analysis and comparison of the mean values of the COPSOQ scale between the group of drivers and the Spanish population. A multivariate analysis was performed using a logistic regression model to explore which psychosocial aspects influenced the degree of job satisfaction, where the dichotomous dependent variable (level of job satisfaction) was assigned two values: 1 = good satisfaction (scores above the 75th percentile) and 0 = low job satisfaction (below the 75th percentile). Predictor or independent variables (*p* < 0.05), point estimate of risk (OR) and 95% confidence interval (CI) were estimated. All analyses were performed in SPSS statistical software, ver. 25.0.

## Results

A total of 601 drivers participated in the study, a higher number than the estimated sample size required. Table 1 shows a socio-demographic description of the sample.

**Table 1:** Sample socio-demographic description.

Variable	*N* %
**Gender**	
Men	97%
Women	3%
Age in years (average, standard deviation)	43.9 (8.3)
Length of time on the driver’s license (average, standard deviation)	22.6 (8.8)
Years of professional experience (average, standard deviation)	15.8 (9.1)
**Degree of general job satisfaction (SG)**	
Very unsatisfied	8.7%
Dissatisfied	9.8%
Little unsatisfied	9.2%
Neutral	17.4%
Little satisfied	18.9%
Satisfied	27.9%
Very satisfied	8.1%

There is a clear predominance of the male gender, with average dates of 43.9 years old, 22.6 years of driving license holding and 15.8 years of seniority in the profession. Regarding the degree of general job satisfaction, 54.9% were satisfied with their job, with values of not very satisfied, satisfied and very satisfied. Similarly, extrinsic satisfaction was 55.7% and intrinsic satisfaction 53.9%.

The items that most influence a lower overall satisfaction are (in order from highest to lowest weight): salary, promotion possibilities in the company, level of attention and participation in the company, the way the company is managed, and the working hours (Table 2).

**Table 2:** Job satisfaction items results.

**Items**	**Mean (SD)**
(1) Psychical conditions	4.65 (1.56)
(2) Freedom method work	4.56 (1.67)
(3) Co-workers	5.14 (1.50)
(4) Social recognition	4.32 (1.77)
(5) Immediate superior	4.60 (1.66)
(6) Level of responsibility	4.95 (1.53)
(7) Salary	3.78 (1.88)
(8) Possibility of using personal skills	4.63 (1.63)
(9) Relationship between company and workers	4.37 (1.73)
(10) Possibility for advancement in the company	3.99 (1.76)
(11) Management in the company	4.15 (1.77)
(12) Level of attention and participation	4.03 (1.74)
(13) Working hours	4.23 (1.95)
(14) Varied work	4.57 (1.67)
(15) Job stability	4.64 (1.93)

**Figure 3:**
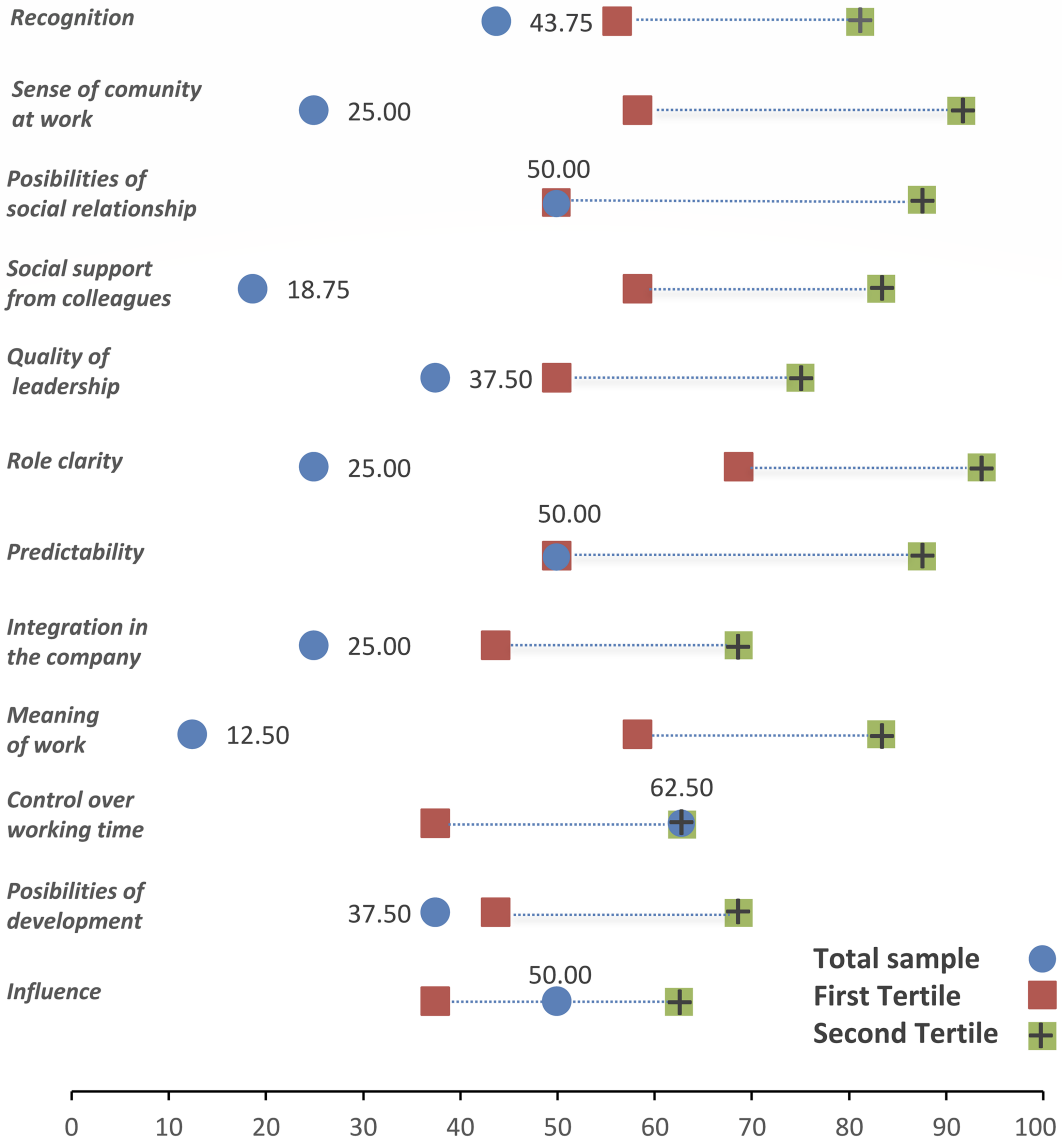
Description of the positive psychosocial dimensions.

**Figure 4:**
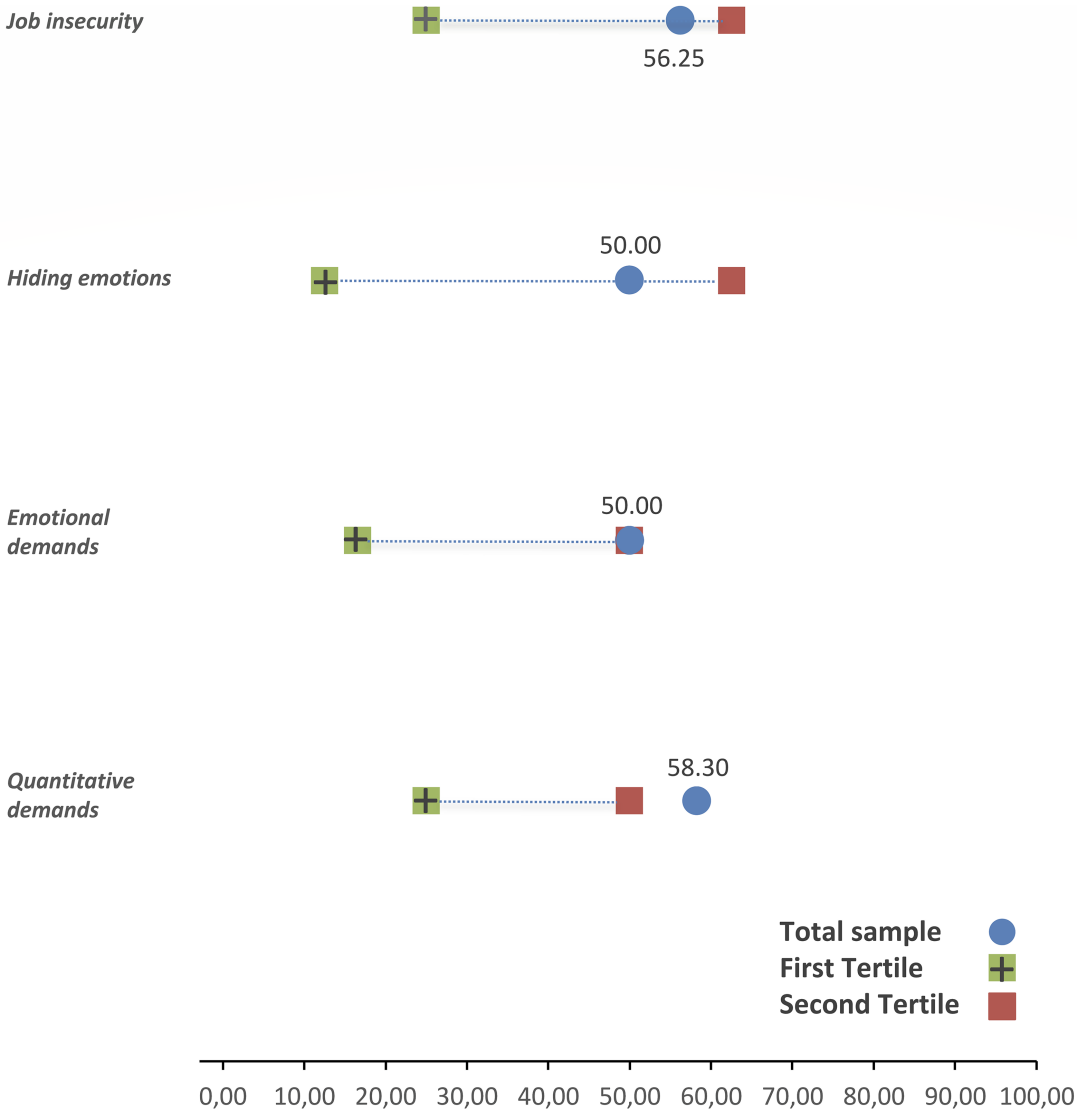
Description of the negative psychosocial dimensions.

Figures 3, 4 show the values of the positive and negative psychosocial scales. A green square shows the upper (lower) tertile for positive (negative) scales, and a red square shows the lower (upper) tertile for positive (negative) scales corresponding to the Spanish salaried population. The mean values of psychosocial scales are represented by a blue dot, so that a blue dot between the two squares represents an intermediate situation. We have a favorable situation if the blue dot is above the green square in positive scales or below the green square in negative scales. And finally, an unfavorable situation arises if the blue dot is above the red square in negative scales or below the red square in positive scales. Except for control over working time (favorable situation) and influence (intermediate situation), participants are in an unfavorable situation in all positive scales (Figure 3). Concerning negative scales (Figure 4), participants are in an unfavorable situation for quantitative and emotional demands, and no negative scales show a favorable situation.

**Figure 5:**
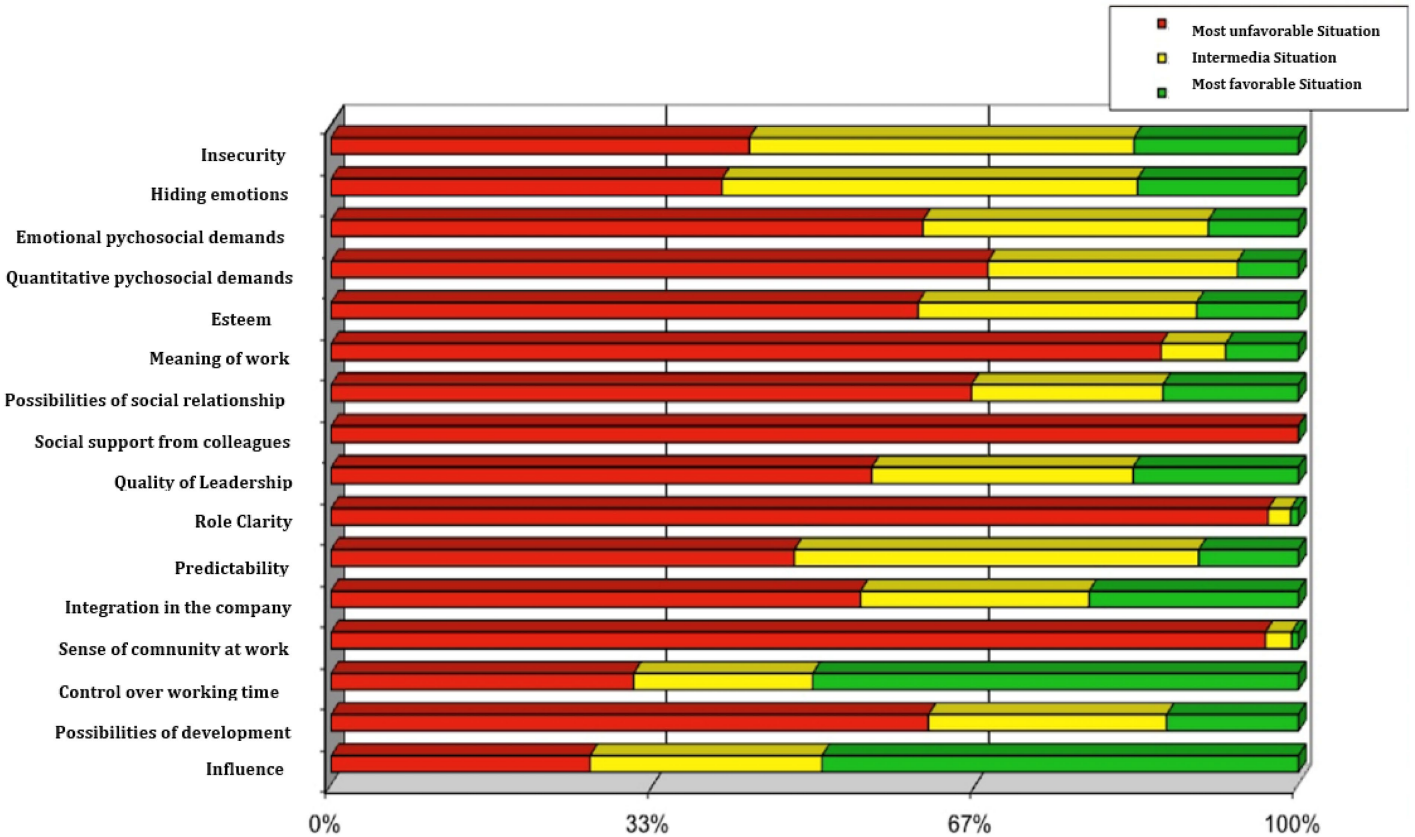
Level of psychosocial risk of professional drivers.

A way of expressing the magnitude of psychosocial risk has been proposed using the so-called ISTAS traffic light (Moncada-Lluís et al., 2008). The exact population tertiles for the different dimensions are established and a bar chart shows the percentage of people in our sample who are in the “most unhealthy situation” (red), “intermediate situation” (yellow) and “most healthy situation” (green). The values for the professional drivers are shown in Figure 5. The most unfavorable psychosocial scales compared to the Spanish reference population are, in this order: Social support from colleagues, Role clarity, Sense of community at work, Meaning of work, and level of quantitative psychological demands.

Table 3 shows the statistical association between the psychosocial dimensions and the degree of job satisfaction, with the exception of the variable support and level of social relations, where no significant association was observed.

**Table 3:** Statistical association between psychosocial factors (^*^) and level of job satisfaction (^**^).

Level job satisfaction^**^	Psychosocial dimensions^*^	Chi-square	*p*
	Quantitative	78.6	<0.001
	Emotional	51.8	<0.001
	Hiding emotions	38.6	<0.001
	Influence at work/Good	20.0	<0.001
	Possibilities for development/Good	32.1	<0.001
Good satisfaction (>75 percentile)	Control over working time	31.8	<0.001
	Meaning of work	25.9	<0.001
	Integration in the company	146.5	<0.001
	Social support	–	NS
Bad satisfaction (<75 percentile)	Social relation	–	NS
	Sense of community at work	23.1	<0.001
	Predictability	146.1	<0.001
	Role clarity	8.4	<0.05
	Quality of leadership	13.5	<0.001
	Recognition	182	<0.001
	Job insecurity	43.6	<0.001

* Tertiles of the psychosocial risk (First, second and third tertile).

** Good and bad job satisfaction.

In order to determine the weight of psychosocial factors on the degree of job satisfaction, a logistic regression model was performed, adjusting for age, gender, length of driving license and years of professional experience. The results are shown in Table 4, which shows the significant variables included in the model, the estimated risk expressed by the OR and the corresponding 95% confidence intervals. The variables that have a significant influence are: sense of community at work, integration in the company, social relation, and recognition. In addition to job insecurity and levels of psychological demands, both quantitatively and emotionally. It is important to remember the interpretation of the “positive” psychosocial factors, where a high score is beneficial, and a low score is detrimental; and in the case of the “negative” ones, they are interpreted in the opposite way, a low score is equivalent to a good situation, and a high score would imply a psychosocial risk.

**Table 4:** Multivariate analysis adjusted by Odds Ratio (OR) and 95% confidence intervals for positive and negative psychosocial factors and the level of job satisfaction.

Independent variables	Positive psychosocial factors^*^			Negative psychosocial factors^*^		
	OR	IC (95%)	*p* ^**^	OR	IC (95%)	*p* ^**^
Sense of community at work	1.01	1.003–1.033	<0.05	–	–	–
Integration in the company	1.02	1.011–1.031	<0.05	–	–	–
Social relation	1.01	1.002–1.018	<0.05	–	–	–
Recognition	1.06	1.040–1.081	<0.05	–	–	–
Level of quantitative demands	–	–	–	0.96	0.949–0.975	<0.05
Level of emotional demands	–	–	–	0.98	0.972–0.993	<0.05
Job Insecurity				1.02	1.016–1.036	<0.05

* Model adjusted for age, gender, length of time on the drivers license and years of professional experience.

** Only significant variables are shown with a *p* < 0.05.

## Discussion

Our study of 601 workers from 12 workplaces provides good support for the study of the relationships between job satisfaction and psychosocial risk factors of professional drivers. This study has used one of the recognized methods for assessing the psychosocial environment of workers (COPSOQ), proposed by the Danish National Institute for Occupational Health (Kristensen et al., 2005). The aim is to show the relationship between psychosocial factors and job satisfaction of professional drivers in Spain. In addition, it allows a comparison of the psychosocial scales of drivers with the average values of the Spanish salaried population taken as a reference (Moncada-Lluís et al., 2008). In general, professional drivers in Spain do not present a favorable situation in any of the psychosocial scales.

Multiple studies have evaluated the conditions of professional drivers and the factors of their activity that can bring serious consequences on the physical and psychological health of these workers (Kresal et al., 2017; Useche et al., 2018, 2021; Arias-Meléndez et al., 2021).

The average age of the drivers (43.9 years) was similar to the Spanish population (43.3 years) according to the latest provisional data from the National Institute of Statistics (INE, 2016). There is a clear predominance of the male gender, which is in line with the norm for this unique profession. In a study carried out in Spain to assess working conditions in public road transport at the National School of Occupational Medicine, the mean age of the drivers was statistically higher than that of the reference population (mean difference = 3.6 with a CI of 3.1–4.1). Also, drivers perceived with more risk than the reference population that their work affected their health (OR = 1.57 with a CI 1.38–1.79; Ordaz-Castillo and Maqueda-Blasco, 2014).

We observe that many psychosocial positive scales are below the lower tertile (possibility of development, sense of work, integration in the company, role clarity, quality of leadership, social support, sense of group and level of esteem). This would indicate that, on average, professional drivers find themselves in a more unfavorable psychosocial environment than the average population of Spanish workers (Moncada-Lluís et al., 2008).

Figure 5 shows the psychosocial scales with the greatest and least weight in the psychosocial risk of professional drivers. On the one hand we have social support, role clarity, sense of work, group feeling, possibility of development and level of psychological demands as those related to greater risk; and on the other hand, the level of influence at work and control over working time associated with lower risk. A recent study has examined the association between bus drivers’ psychosocial factors and risky driving behaviors. The results of this study suggest that stress-related working conditions (job strain, social support and effort/reward imbalance) are relevant predictors of risky driving in Bus Rapid Transport drivers, and that fatigue is the mechanism linking other types of stress related to risky driving behaviors links other work condition-related stress (job strain and low social support) to risky driving (Useche et al., 2017).

Studies on healthcare workers showed many more favorable scales: group feeling, social relations, integration in the company, sense of work, development possibilities and influence at work (García-Rodríguez et al., 2015; Miller et al., 2018) despite suffering from stress and poor organizational support from their supervisors (Hamdan-Mansour et al., 2011). Other similar studies carried out on healthcare personnel show similar favorable results related to psychosocial risks: group feeling, social relations, integration in the company, sense of work, possibilities for development and influence at work, work flexibility, freedom of decision, rest practices and meal breaks (Rodriguez-Alonso et al., 2017). This would indicate that workers in the health sector, which is a sector of high work pressure, would have a less harmful psychosocial environment than professional drivers, a curious fact that is difficult to justify. It is possible that health workers are much more rewarded in the development of their work activity than professional drivers.

In relation to job satisfaction, professional drivers perform worse than other groups of workers. For example, workers in the administrative sector had a level of satisfaction close to 80% (Montero et al., 2015); in the health sector, 83.9% (Rodriguez-Alonso et al., 2017), a fact that could be due to the general conditions of their work and the psychosocial environment of their activity. In our study we observed a significant association between most of the psychosocial dimensions and the degree of job satisfaction (Table 4), with the exception of support and level of social relationships. A similar behavior occurs in the group of construction workers (Sáenz, 2016); however, in other activities of evident social and affective involvement, such as the group of workers in oncology units, social support, level of social relationships and quality of leadership are very important aspects in their degree of job satisfaction (Rivera-Rojas et al., 2021).

The association between job satisfaction and psychosocial work environment factors is maintained in multivariate models after adjusting for age, gender, and driving experience. In the multivariate analysis (Table 4), it is observed that seven psychosocial scales remain in the model. This result is very promising and would support the fact that job satisfaction is strongly influenced by the psychosocial environment of professional drivers. It is in line with the fact that job satisfaction should be considered as an emotional response to multiple factors related to the worker’s perception of their working conditions and job stress; and not only to the physical, ergonomic and salary aspects of the work activity (Agnieszka et al., 2018; Sureda et al., 2018).

Although we use multivariate regression models to adjust for these factors, other residual confounders may still exist. However, it should be noted that some of these factors can be considered as mediators of the association.

Our work has been limited to assessing psychosocial factors related to working conditions and the working environment. We are aware that other factors related to health status that could influence the degree of job satisfaction have not been assessed. In this sense, it is important to promote extensive multidisciplinary research that addresses all these aspects together.

In conclusion, professional drivers carry out a work activity with specific characteristics which are difficult to assess and which have been linked to health problems, especially those arising from physical and ergonomic aspects. This study aims to provide evidence that this work activity is also influenced by psychosocial risk factors, which not only influence the degree of job satisfaction, but may also have a negative effect on the health of professional drivers.

## Data availability statement

The raw data supporting the conclusions of this article will be made available by the authors, without undue reservation.

## Author contributions

AG-R and EM-G: conceptualization, supervision, and writing—original draft. VH-R and MG-B: data curation and formal analysis. AG-R and MG-B: methodology. MG-B: validation. VH-R and EM-G: writing—review and editing. All authors contributed to the article and approved the submitted version.

## Funding

The APC was funded by Universidad de Málaga/CBUA.

## Conflict of interest

The authors declare that the research was conducted in the absence of any commercial or financial relationships that could be construed as a potential conflict of interest.

## Publisher’s note

All claims expressed in this article are solely those of the authors and do not necessarily represent those of their affiliated organizations, or those of the publisher, the editors and the reviewers. Any product that may be evaluated in this article, or claim that may be made by its manufacturer, is not guaranteed or endorsed by the publisher.

## Footnotes

^1^ http://copsoq.istas21.net
